# Pleiotropic effects of antibiotics on T cell metabolism and T cell-mediated immunity

**DOI:** 10.3389/fmicb.2022.975436

**Published:** 2022-10-18

**Authors:** Tobias Franz, Jonas Negele, Philipp Bruno, Martin Böttcher, Marisa Mitchell-Flack, Lea Reemts, Anna Krone, Dimitrios Mougiakakos, Andreas J. Müller, Andreas E. Zautner, Sascha Kahlfuss

**Affiliations:** ^1^Medical Faculty, Institute of Molecular and Clinical Immunology, Otto-von-Guericke University Magdeburg, Magdeburg, Germany; ^2^Department of Hematology and Oncology, University Hospital Magdeburg, Otto-von-Guericke University Magdeburg, Magdeburg, Germany; ^3^Medical Faculty, Health Campus Immunology, Infectiology and Inflammation (GCI-3), Otto-von-Guericke University Magdeburg, Magdeburg, Germany; ^4^Department of Oncology, The Bloomberg∼Kimmel Institute for Cancer Immunotherapy, Johns Hopkins University School of Medicine, Baltimore, MD, United States; ^5^Medical Faculty, Institute of Medical Microbiology and Hospital Hygiene, Otto-von-Guericke University Magdeburg, Magdeburg, Germany; ^6^Center for Health and Medical Prevention (CHaMP), Otto-von-Guericke University Magdeburg, Magdeburg, Germany

**Keywords:** antibiotics, metabolism, bacteria, mitochondria, host-pathogen, T cells

## Abstract

T cells orchestrate adaptive and innate immune responses against pathogens and transformed cells. However, T cells are also the main adaptive effector cells that mediate allergic and autoimmune reactions. Within the last few years, it has become abundantly clear that activation, differentiation, effector function, and environmental adaptation of T cells is closely linked to their energy metabolism. Beyond the provision of energy equivalents, metabolic pathways in T cells generate building blocks required for clonal expansion. Furthermore, metabolic intermediates directly serve as a source for epigenetic gene regulation by histone and DNA modification mechanisms. To date, several antibiotics were demonstrated to modulate the metabolism of T cells especially by altering mitochondrial function. Here, we set out to systematically review current evidence about how beta-lactam antibiotics, macrolides, fluoroquinolones, tetracyclines, oxazolidinones, nitroimidazoles, and amphenicols alter the metabolism and effector functions of CD4^+^ T helper cell populations and CD8^+^ T cells *in vitro* and *in vivo*. Based on this evidence, we have developed an overview on how the use of these antibiotics may be beneficial or detrimental in T cell-mediated physiological and pathogenic immune responses, such as allergic and autoimmune diseases, by altering the metabolism of different T cell populations.

## Introduction

The discovery of antibiotics as antibacterial substances was one of the most significant scientific findings in the last century. Since then, several antibiotics targeting specific bacteria have been developed. Conversely, antibiotics were also reported to affect the host immune system by modulating adaptive immune cells such as T cells. In this context, especially beta-lactam antibiotics, macrolides, fluoroquinolones, tetracyclines, oxazolidinones, nitroimidazoles, and amphenicols were demonstrated to interfere with T cell metabolism and effector functions.

**FIGURE 1:**
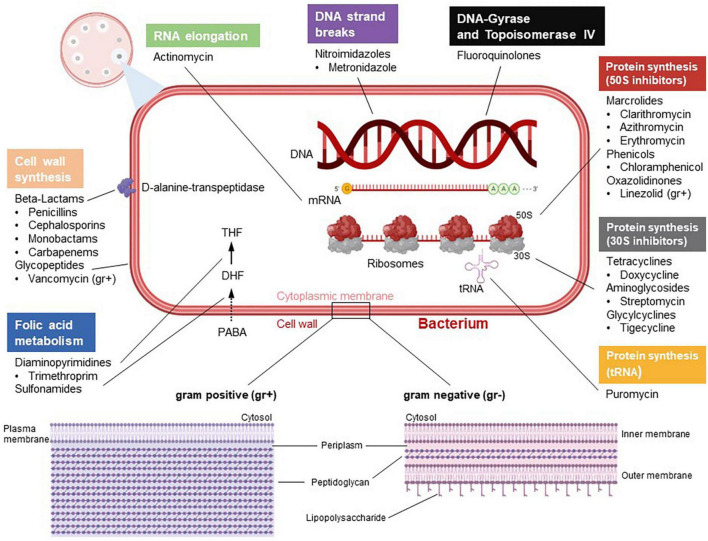
Categorization and mode of actions of antibiotics. According to their barrier structure, bacteria can be divided into gram positive (gr +) and gram negative (gr–). Antibiotics differ in terms of their mechanisms. Some antibiotics target the bacterial cell wall. Beta-lactam antibiotics directly inhibit the enzyme D-alanine-transpeptidase, which impedes cross-linking of building blocks in bacterial murein synthesis. Glycopeptides such as vancomycin, which only attack gram positive bacteria, inhibit bacterial cell wall synthesis by complex formation with murein components. Diaminopyrimidines, e.g., trimethoprim, inhibiting the enzyme dihydrofolate reductase, and sulfonamides, which target the diydropteroatsynthase, inhibit bacterial tetraydrofolate (THF) synthesis and, hence, DNA synthesis. Fluoroquinolones negatively regulate the bacterial enzymes topoisomerase II (gyrase) and topoisomerase IV, which impairs DNA replication. Actinomycin has cytostatic capabilities by intercalating into DNA and thus inhibiting RNA elongation. Moreover, certain antibiotics impede bacterial protein synthesis through binding to 70S ribosomes. In this context, macrolides, phenicols and oxazolidiones inhibit the large 50S subunit, whereas tetracyclins, aminoglycosides and glycylcylins are potent 30S inhibitors. In addition, drugs like puromycin interfere with tRNA function and thus represent another mechanism of protein synthesis inhibition. Metronidazol induces DNA strand breaks. The figure was created with BioRender.com.

Naïve T cells in secondary lymphoid organs predominantly perform oxidative phosphorylation (OXPHOS). However, T cell activation, for example by recognition of bacterial, viral and parasitic antigens, induces the expression of several genes including genes encoding for the rate-limiting enzymes of glycolysis but also of the electron transport chain, which cumulates in a switch to aerobic glycolysis (Buck et al., 2017). Importantly, expression of glycolysis genes is to a significant degree directly regulated through T cell receptor (TCR)-mediated store-operated calcium entry (SOCE) and mediated by calcium-dependent transcription factors such as nuclear factor of activated T cells (NFAT) family members (Vaeth et al., 2017, 2020; Kahlfuss et al., 2020). The switch of activated T cells from OXPHOS to glycolysis is known as metabolic reprogramming. Following activation, T cells do not utilize glycolysis simply to generate energy equivalents but rather to form building blocks such as amino acids and nucleotides for consecutive proliferation. The latter is crucial as T cells have to acquire a critical amount of biomass in preparation for prospective cell divisions during clonal expansion. Importantly, after an infection is cleared, a minor fraction of T cells differentiate into memory T cells, which switch back to OXPHOS as their low division rate does not require the generation of a significant amount of metabolic building blocks. Antibiotics were shown to partially interfere with the activation, metabolism, differentiation, and effector functions of T cells. Based upon their structure and/or mode of action, antibiotics are divided into different groups (Hutchings et al., 2019; Figure 1).

In this context, beta-lactam antibiotics including penicillins, cephalosporins, carbapenems, and monobactams contain a beta-lactam ring, that inhibits the bacterial D-alanyl-D-alanine carboxypeptidase often referred to as DD-transpeptidase or functionally paraphrased as penicillin-binding protein (PBP) (Hutchings et al., 2019). Inhibition of the DD-transpeptidase impedes cell wall synthesis during bacterial proliferation by binary fission. The lead substance penicillin shows efficiency against Gram-positive bacteria such as *Streptococcus* species and *Clostridium perfringens* but also against Gram-negative germs such as *Neisseria* species and *Spirochaete*. Furthermore, penicillinase-resistant penicillins (second-generation penicillins) such as flucloxacillin are frequently used against *Staphylococci* that express penicillinases. In addition, extended-spectrum penicillins such as aminopenicillins, carboxypenicillins or ureidopenicllins are efficient against several Gram-negative bacteria. There is also a gradation in the activity spectrum of cephalosporins. While first- and second-generation cephalosporins show relatively good activity against Gram-positive pathogens, the extended-spectrum cephalosporins of the third and fourth generation are more effective against Gram-negative rods. Carbapenems, e.g., imipenem and meropenem, are considered broad-spectrum antibiotics and should be used judiciously to prevent the generation of resistant bacterial strains. The only monobactam in clinical use, aztreonam, has found its niche in the treatment of bacteria expressing metallo-beta-lactamases as it is not hydrolyzed by them.

Macrolides prevent bacterial protein synthesis by interfering with 50S ribosome subunits. Macrolides are used to treat Gram-positive bacteria such as, e.g., *Streptococcus pneumonia* but also Gram-negative bacteria, e.g., *Haemophilus influenza*. Macrolides are frequently used as an alternative treatment option for patients suffering from allergy against beta-lactam antibiotics (Hutchings et al., 2019).

Fluoroquinolones act as inhibitors of the bacterial topoisomerase II and IV. By inhibiting DNA despiralization, supercoil relaxation, and DNA unlinking after DNA replication, they interfere with DNA synthesis (Figure 1). Fluoroquinolones can be divided into four generations (Hutchings et al., 2019). While second generation fluoroquinolones feature higher efficiency against Gram-negative bacteria including non-fermenter, third and fourth generation fluoroquinolones are used in the treatment of Gram-positive and bacteria without cell wall as well as Gram-positive anaerobic bacteria such as *Peptostreptococci*.

Tetracyclines inhibit protein synthesis by interaction with the ribosomal 30S subunit (Figure 1). Tetracyclines are frequently used to treat infections in patients, who are allergic to beta-lactam antibiotics and macrolides. As a common structural characteristic, all tetracyclines share a linear fused tetracyclic nucleus with different functional groups. Clinically, tetracyclines are effective in the treatment of Gram-negative intracellular bacteria such as *Chlamydia* or *Rickettsia*.

Linezolid is used against infections caused by Gram-positive bacteria including penicillin-resistant streptococci, vancomycin-resistant enterococci (VRE), and methicillin-resistant *Staphylococcus aureus* (MRSA) (Figure 1). Linezolid is a bacterial protein synthesis inhibitor that prevents translation by interfering with the formation of the initiation complex, which is composed of the 30S and 50S subunits of bacterial ribosomes, N-formyl-methionyl-tRNA, and mRNA. Metronidazole is a nitroimidazole used to treat *Trichomonas vaginalis*, *Giardiasis*, or *C. difficile* colitis as it inhibits nucleic acid synthesis by forming radicals, which disrupts DNA structure.

Chloramphenicol, belonging to the amphenicols, reversibly binds to the catalytic site of the 50S subunit peptidyl transferase of bacterial 70S ribosomes and is effective against *Staphylococcus aureus, Streptococcus pneumoniae*, and *Escherichia coli*.

CD8^+^ T cells mediate immunity against viruses but also against other infectious pathogens and tumors. CD4^+^ T cells can differentiate into several T helper (Th) cell populations such as Th1, Th2, Th17, and regulatory T cells (T_regs_). Th1 cells provide cell-mediated immunity against intracellular bacteria and viruses, while Th2 cells are involved in humoral immune response against extracellular bacteria and parasites (Dong, 2021). Th17 cells regulate cell-mediated immunity against extracellular pathogens (bacteria and parasites) and fungi. However, Th1, Th2, and Th17 cells are also involved in mediating autoimmune and allergic reactions (Skapenko et al., 2005; Zhao et al., 2013; León and Ballesteros-Tato, 2021). T_regs_ negatively regulate CD8^+^ T cells and all other CD4^+^ Th cell populations to prevent overwhelming and/or long-lasting immune reactions. It should be taken into consideration that other Th cell populations such as the recently described Th9 cells exist, and that the different Th cell populations especially *in vivo* are assumed to represent rather a continuum with intersubset plasticity than distinct lineages. Interestingly, it was reported that the differentiation of T cells into distinct T cell populations is accompanied by the usage of specific metabolic pathways. *In vivo*, Th cells must metabolically adapt to their environment. Thus, T cell metabolism is significantly involved in the activation, differentiation, and effector function of CD8^+^ cytotoxic T cells (CTLs) and CD4^+^ Th cells. However, how antibiotics exert pleiotropic effects on T cell metabolism and function has not yet been comprehensively reviewed.

Therefore, here we have set out to systematically discuss the current evidence how beta-lactam antibiotics, macrolides, fluoroquinolones, tetracyclines, oxazolidinones, nitroimidazoles, and amphenicols interfere with T cell metabolism and thus T cell-mediated immunity, and what therapeutic potential this may provide for autoimmune and allergic diseases independent of their primary indication in bacterial infections.

## Antibiotics influence CD4^+^ T helper cell function

**FIGURE 2:**
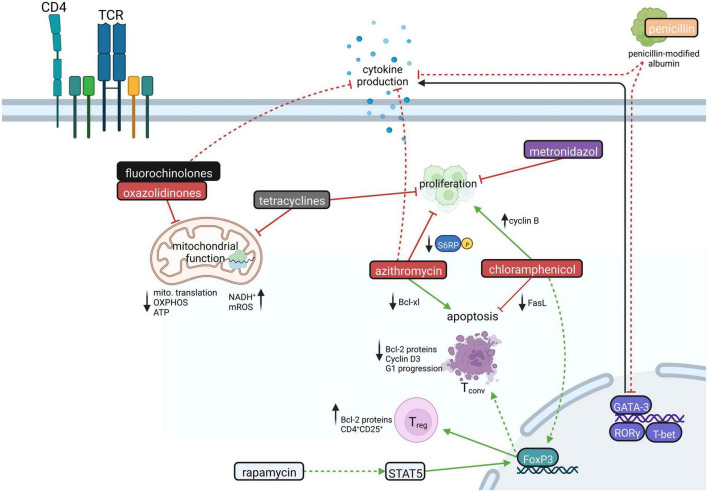
The influence of antibiotics on CD4^+^ T cells. CD4^+^ T cell proliferation is inhibited by tetracycline, azithromycin, and metronidazole. Chloramphenicol, in turn, forces proliferation by increasing cyclin B expression. Azithromycin, favoring the downregulation of the antiapoptotic Bcl-xl protein, increases CD4^+^ T cell apoptosis, whereas chloramphenicol hampers apoptosis by downregulating Fas ligand. Antibiotics like fluorochinolones, tetracyclines, and oxazolidinones inhibit mitochondrial function leading to decreased mitochondrial translation and ATP production via OXPHOS and increased levels of NADH^+^ and mitochondrial reactive oxygen species (mROS). Moreover, antibiotics influence important transcription factors in various ways. GATA-3 is downregulated by penicillin, while chloramphenicol treatment leads to FoxP3 upregulation. Rapamycin likewise induces the upregulation of FoxP3, which promotes the generation of CD4^+^CD25^+^ regulatory T cells (T_reg_) while conventional T cells (T_conv_) are driven to apoptosis. Moreover, fluorochinolones, azithromycin, and penicillin could lead to decreased production of CD4^+^ T cell effector cytokines. Created with BioRender.com.

Aside from antimicrobial activity, several antibiotics have immunomodulatory capacities by altering the function of CD4^+^ T cells (Figure 2). In general, antibiotics have been reported to downregulate proinflammatory cytokine responses including IL-1β, IL-6, IL-8, and TNF-α (Bailly et al., 1990; Dalhoff and Shalit, 2003; Williams et al., 2005; Shams et al., 2021), to alleviate inflammatory processes by inducing IL-10 (Becker et al., 2016; Seifert et al., 2018) or to directly affect the development and/or function of Th1, Th2 and Th17 cells (Williams et al., 2005; Matsui et al., 2016, 2019).

Linezolid, a ribosomal-targeting antibiotic (RAbo) belonging to the substance group of oxazolidinones, inhibits bacterial protein synthesis by targeting the formation of bacterial 70S ribosomes, particularly affecting Gram-positive bacteria. According to the endosymbiont theory, mitochondria possess prokaryotic origin (Esposti et al., 2014), and therefore structural similarities between bacterial and mitochondrial ribosomes are conserved (Kondo and Westhof, 2008; Esposti et al., 2014). Linezolid and other RAbos such as tigecycline and thiamphenicol show pleiotropic effects on host cell physiology by inhibiting mitochondrial translation (Zhang et al., 2005; McKee et al., 2006; Moullan et al., 2015; Almeida et al., 2021), which was reported to impair T cell function (Almeida et al., 2021; Figure 2). Especially, linezolid was shown to inhibit Th cell effector functions including IFN-γ, IL-13 and IL-17 production, while viability of Th1, Th2 and Th17 cells appeared unaffected (Almeida et al., 2021). Mechanistically, ribosome-targeting antibiotics inhibit mitochondrial translation of electron transport chain complexes (ETCs) by blocking the peptidyl transferase center of mitochondrial ribosomes (Leach et al., 2007; Almeida et al., 2021). This results in an imbalance between nuclear- and mitochondrial-encoded ETC subunits (Houtkooper et al., 2013), which compresses the integrity of the ETC and subsequently disrupts OXPHOS (Almeida et al., 2021). As a result, OXPHOS-derived ATP production is impaired and nicotinamide adenine dinucleotide (NAD^+^) regeneration in differentiating T cells appears compromised, leading to impaired Th cell function and cytokine production, particularly in Th17 cells (Almeida et al., 2021). Whether and to which extent this mechanism of action also applies to other Th cell populations besides Th17 cells is currently under investigation. In addition, linezolid treatment interferes with the expression of glycolysis genes (Almeida et al., 2021), which are mainly involved in TCR-induced T cell activation.

Fluoroquinolones were shown to exert immunomodulatory properties by suppressing the production of proinflammatory cytokines including IL-1β, IL-6, TNF-α. Here, various underlying mechanisms were proposed, including inhibition of phosphodiesterases and transcription factors, such as AP-1, NF-AT, NF-IL-6, and NF-κB (Khan et al., 2000; Dalhoff and Shalit, 2003; Dalhoff, 2005; Zhang and Ward, 2008; Ogino et al., 2009; Zusso et al., 2019). However, for T cells, there are numerous contradictory reports that ciprofloxacin either activates or inhibits T cell activation-induced gene expression, such as IFN-γ, TNF-α, IL-4, and IL-2 (Stünkel et al., 1991; Williams et al., 2005; Katsuno et al., 2006; Zhang and Ward, 2008; Kamiński et al., 2010). The latter regulates T cell growth and effector functions (Kamiński et al., 2010; Assar et al., 2021; Figure 2). Comparable to RAbos, ciprofloxacin evokes loss of mitochondrial DNA, which compromises mitochondrial function and suppresses cell growth in pre-activated human T cells (Kamiński et al., 2010). Mechanistically, prolonged ciprofloxacin treatment was shown to lead to impaired activity of the mtDNA-encoded complex I of the ETC, which reduces T-cell activation-induced ROS production and thereby enhances activation of the redox-dependent transcription factors NF-κB and AP-1.

Penicillin G was shown to exhibit anti-inflammatory properties by impairing *GATA3*, *TBX21*, *IFNG*, and *IL17A* gene expression in T cells (Shams et al., 2021; Figure 2). However, beta-lactam antibiotics also have opposing effects on immune-related gene expression in T cells: This is reflected by the fact that cefuroxime was shown to downregulate genes related to Th2 and T_*reg*_ differentiation, while ampicillin was reported to upregulate these genes (Christie et al., 1987; Mor and Cohen, 2013). Mechanistically, beta-lactams such as penicillin covalently bind to serum albumin (Christie et al., 1987; Mor and Cohen, 2013), which can be taken up by T cells. Uptake of beta-lactams bound to albumin may then secondarily alter T cell gene expression (Mor and Cohen, 2013). However, the exact molecular mechanisms of penicillin-modified albumin on gene expression remain unknown.

Upon doxycycline and metronidazole treatment, induction of proliferation-associated signaling pathways was observed (Becker et al., 2016; Figure 2). Doxycycline upregulates proinflammatory signaling pathways in T_regs_ and naive T cells including NF-κB and IL-13 signaling (Becker et al., 2016). *Vice versa*, metronidazole induced an anti-inflammatory expression profile in these cells (Becker et al., 2016).

Chloramphenicol was reported to cause abnormal cellular differentiation in activated T cells *via* overexpression of cyclin B as well as inhibition of activation-induced cell death *via* downregulation of Fas ligand (FasL) expression. In fact, this mechanism also confers to the leukemia-inducing potential of chloramphenicol (Yuan and Shi, 2008; Figure 2). In addition, chloramphenicol treatment was shown to promote differentiation into T_regs_ *via* upregulation of the fate-specific transcription factor FOXP3 (Yuan and Shi, 2008).

Macrolides, such as clarithromycin, erythromycin, azithromycin, and the immunosuppressant rapamycin (sirolimus), which belongs to the same substance group, have been recognized for their immunomodulatory effects (Plewig and Schoepf, 1975; Kudoh et al., 1998; Khan et al., 1999; Zuckerman, 2004; Kanoh and Rubin, 2010; Ratzinger et al., 2014; Zimmermann et al., 2018; Bergström et al., 2019; Weng et al., 2019). This has cumulated in further research on the usage of macrolides for the treatment of chronic inflammatory diseases (Kudoh et al., 1998; Wolter et al., 2002; Gotfried, 2004; Clement et al., 2006; Simpson et al., 2008; Sadreddini et al., 2009; Albert et al., 2011; Koutsoubari et al., 2012; Wong et al., 2012; Brusselle et al., 2013; Zimmermann et al., 2018). CD4^+^ T cells were reported to show suppressed Th1 and Th2 effector cytokine production in a dose-dependent manner in the presence of macrolides (Macleod et al., 2001; Kraft et al., 2002; Uli et al., 2002; Berg et al., 2003; Park et al., 2004; Pukhalsky et al., 2004; Williams et al., 2005; He et al., 2010; Perica et al., 2010; Periæ et al., 2012; Tkalèeviæ et al., 2012; Zimmermann et al., 2018). Furthermore, azithromycin was demonstrated to inhibit CD4^+^ T cell proliferation (Hiwatashi et al., 2011; Ratzinger et al., 2014; Lin et al., 2016) and to promote apoptosis through the modulation of the mammalian Target of Rapamycin (mTOR) (Mizunoe et al., 2004; Ratzinger et al., 2014; Figure 2). Mechanistically, azithromycin, and clarithromycin inhibit phosphorylation of ribosomal S6 protein, a downstream target of mTOR and thereby impair cell growth and proliferation in a manner independent of 12-kDa FK506-and-Rapamycin-binding protein (FKBP12) (Ratzinger et al., 2014). Downstream of mTOR, macrolides inhibit the expression of Bcl-xl, an inhibitor of apoptosis, which makes it likely that the downregulation of antiapoptotic factors is one of the molecular mechanisms underlying how macrolides enhance T cell apoptosis (Mizunoe et al., 2004). Of note, macrolides also exert immunomodulatory effects on other cells of the adaptive and innate immune systems, such as neutrophils and eosinophils (Sugihara, 1997; Wallwork and Coman, 2004; Zimmermann et al., 2018).

## Various effects of antibiotics on proliferation, apoptosis, and effector functions of CD8^+^ T cells and Jurkat T cells

CD8^+^ CTLs eliminate intracellular pathogens and mediate tumor surveillance. To this end, CTLs secrete proinflammatory cytokines, produce and release cytotoxic granules and induce active cell death of infected or transformed cells *via* Fas/FasL interaction (st. Paul and Ohashi, 2020). Compared to CD4^+^ T cells, relatively little is known about if and how antibiotics influence the mitochondrial function and/or metabolism of CTLs.

**FIGURE 3:**
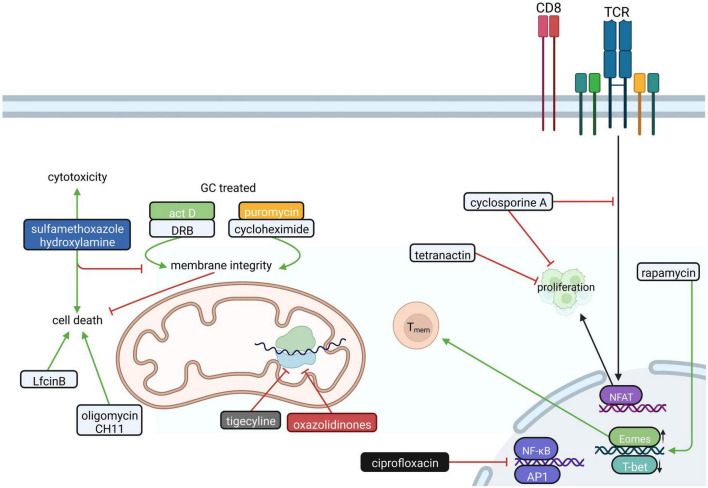
The influence of antibiotics on CD8^+^ T cells. In CD8^+^ T cells antibiotics like tetranactin and cyclosporin A interfere with proliferation by inhibiting the transcription factor NFAT. Additionally, many antibiotics influence mitochondrial function in CD8^+^ T cells. Actinomycin D (Act D), daunorubicin (DRB), cycloheximide and puromycin impair the glucocorticoid (GC)-induced loss of mitochondrial membrane integrity, which prevents cell death in CD4^+^CD8^+^ thymocytes. Sulfamethoxazole hydroxylamine, in turn, amplifies apoptosis and is relevant for CTL-mediated cytotoxicity. Furthermore, the attenuation of the mitochondrial membrane potential by Oligomycin and CH11 or activation of the intrinsic mitochondrial apoptosis pathway by bovine lactoferricin (LfcinB) lead to cell death. Furthermore, oxazolidinones and tigecycline interfere with mitochondrial function in CD8^+^ T cells by inhibiting mitochondrial protein translation. The fluoroquinolone ciprofloxacin suppresses the induction of transcription factors NF-κB and AP-1, while Rapamycin favors to the generation of CD8^+^ T memory cells (T_mem_). Created with BioRender.com.

Beta-lactam antibiotics have been shown to suppress the generation and proliferation of virus-specific CTLs in a dose-dependent manner (Huegin et al., 1986). Furthermore, antibiotics such as tetranactin, a macrotetrolide, and cyclosporine A inhibit CTL proliferation (Callewaert et al., 1988; Kamiński et al., 2010; Figure 3), however the exact mechanism for tetranactin remained elusive in these studies. Cyclosporine A, which inhibits nuclear factor of activated T cells (NFAT), is highly effective in inhibiting the production of IL-2 and in preventing IL-2 receptor expression on CTLs. In addition, ciprofloxacin interferes with the production of IL-2 and IL-4 and shows an immunosuppressive effect on both CD4^+^ and CD8^+^ T cells (Kamiński et al., 2010).

Antibiotics do not only interfere with the proliferation of CD8^+^ T cells but they also influence apoptosis. In this specific context, antibiotics such as actinomycin D, daunorubicin or the translational inhibitors cycloheximide and puromycin were reported to inhibit glucocorticoid-induced apoptosis in CD4^+^CD8^+^ thymocytes in a dose-dependent manner (Dezitter et al., 2011; Figure 3). On the other hand, sulfamethoxazole-hydroxylamine treatment was shown to induce a concentration-dependent decrease of CD8^+^ T cell viability and causes suppression of proliferation *in vitro* (Hess et al., 1999). One could assume that these cytotoxic and immunomodulatory effects of sulfonamide reactive metabolites occur selectively in CD8^+^ cells, since purified human CD4^+^ cells appear to be more resistant to sulfamethoxazole-hydroxylamine-induced apoptosis *in vitro* (Hess et al., 1999). Oligomycin, a frequently used ATP synthase inhibitor, potentiated the proapoptotic effects of the Fas-activating antibody (CH11) in Jurkat T cells. Specifically, CH11 and oligomycin led to a decrease in cellular ROS production by attenuating mitochondrial membrane potential (Tirosh et al., 2003). Another inducer of apoptosis is bovine lactoferricin (LfcinB). LfcinB is a cationic antimicrobial peptide that was reported to induce cell death of Jurkat T cells *via* initial cell membrane damage and consequent intrinsic mitochondrial apoptosis pathways (Mader et al., 2007).

Regarding CD8^+^ T cell effector function, penicillin and streptomycin were shown to accelerate target cell lysis by CTLs (Horai et al., 1982) and sulfamethoxazole-hydroxylamine was found to promote the transcription of mitochondrial iron transporters that are important for CTL-mediated cytotoxicity (Reinhart et al., 2018).

Fever is a common symptom of infection and inflammation, but also in advanced stages of cancer. Activated CD8^+^ T cells exposed to febrile temperature promote metabolic activity and functional capacity by enhancing mitochondrial translation (OSullivan et al., 2021). Tigecycline treatment limited this temperature-induced increase in mitochondrial translation and inhibited OXPHOS (OSullivan et al., 2021; Figure 3). In this context, tigecycline attenuated the antitumor response of 39°C primed CD8^+^ T cells in leukemic mice (OSullivan et al., 2021).

## Rapamycin strongly interferes with CD4^+^ and CD8^+^ T cell immunity

Macrolides, such as clarithromycin, erythromycin, and azithromycin, are known for their immunomodulatory effects. The macrolide rapamycin is widely used for the prevention of transplant rejection. As rapamycin was shown to have significant impact on T cell metabolism and effector function, it is discussed here in a separate section.

Rapamycin (sirolimus), discovered in the 1970s on the Easter islands (Fowler, 2014; Lamming, 2016), is produced by the actinomycete *Streptomyces hygroscopicus* (Shan et al., 2014), and has strong immunosuppressive/immunomodulatory capabilities (Shan et al., 2014; Stallone et al., 2016; Figures 2, 3). Clinically, rapamycin is used in order to prevent graft rejection after transplantation (Shan et al., 2014; Bergström et al., 2019; Scheurer et al., 2020) in combination with calcineurin inhibitors (Ehx et al., 2021) and glucocorticoids (Zheng et al., 2007).

Mechanistically, rapamycin binds to the intracellular FKBP12 (Battaglia et al., 2006; Fowler, 2014; Stallone et al., 2016; Bergström et al., 2019), which promotes complex formation, raptor-association, structural changes (Bergström et al., 2019) and results in the inhibition of the mammalian target of rapamycin complex 1 (mTORC1) (Fowler, 2014; Lamming, 2016; Bergström et al., 2019; Schreiber et al., 2019; Scheurer et al., 2020). In addition, rapamycin is capable of inhibiting mTORC2 (Chi, 2012; Lamming, 2016; Schreiber et al., 2019; Scheurer et al., 2020). mTOR proteins are cytoplasmic serine/threonine protein kinases that belong to the phosphoinositide 3-kinase (PI3K)-related family, which act as a key integrator of nutrient uptake, immune signaling, growth signals, and other environmental input signals. Thereby, mTOR factors regulate metabolism, cell cycle, protein synthesis, and cell growth of T cells (Battaglia et al., 2006; Zheng et al., 2007; Chi, 2012; Fowler, 2014; Shan et al., 2014; Lamming, 2016; Stallone et al., 2016; Scheurer et al., 2020). Strikingly, mTOR-deficient T cells are unable to differentiate toward Th1, Th2, and Th17 cells upon TCR stimulation, as they lack the activation of several important lineage-determining transcription factors such as STAT4 (Th1) STAT6 (Th2) or STAT3 (Th17) (Chi, 2012; Geltink et al., 2018). Rapamycin furthermore interferes with cell cycle progression by mediating an arrest of T effector cells in the G1 phase (Allen et al., 2004; Zheng et al., 2007; Chi, 2012; Stallone et al., 2016), which prevents T cell proliferation (Battaglia et al., 2006).

Furthermore, inhibition of PI3K through PTEN or mTOR inhibition by rapamycin was shown to induce FOXP3 expression and thus T_*reg*_ differentiation (Allen et al., 2004; Zeiser et al., 2008; Chi, 2012; Dikiy et al., 2021; Figure 2). In line with this, rapamycin-treated human CD4^+^CD25^+^ T cells proliferated less but showed higher frequencies of FOXP3^+^ cells with suppressive function (Tresoldi et al., 2011). Conversely, rapamycin was shown to decrease CD4^+^CD25^–^ effector T cell expansion but not CD4^+^CD25^+^FOXP3^+^ T_*reg*_ proliferation (Battaglia et al., 2005, 2006; Strauss et al., 2009). In clinical trials, rapamycin treated patients showed increased T_*reg*_ counts (Shan et al., 2014). In addition, in the presence of rapamycin T_regs_ failed to expand to Th17 cells (Tresoldi et al., 2011). Further, Rapamycin interferes with the metabolism of iTregs as it induces metabolic reprogramming of Tregs leading to decreased glucose metabolism and shifting toward enhanced mitochondrial metabolism including increased OXPHOS and FAO (Chen X. et al., 2021). This specific metabolic profile is unique to Tregs (Geltink et al., 2018) which may reveal the interplay between metabolism and Treg differentiation. Together, these findings indicate that rapamycin fosters T_*reg*_ differentiation (Bergström et al., 2019) by interfering with the Akt-mTORC1 pathway that is crucial for T_*reg*_ differentiation and function (Haxhinasto et al., 2008). However, mTOR is also strongly involved in the regulation of activation and anergy of T cells by signaling cascades leading to phosphorylation of the downstream mTOR target S6 kinase 1 (Zheng et al., 2007). It has been shown that rapamycin is capable of inducing anergy in T cells (Zheng et al., 2007), even in the presence of CD28 costimulation (Allen et al., 2004; Zheng et al., 2007; Haxhinasto et al., 2008; Chi, 2012; Stallone et al., 2016). Besides induction of anergy *in vitro*, rapamycin was also reported to induce anergy *in vivo* (Zheng et al., 2007). However, cell cycle arrest in G1 by usage of sanglifehrin A (SFA) (Allen et al., 2004; Zheng et al., 2007) or inhibiting T cell proliferation alone is not sufficient to induce anergy (Powell and Delgoffe, 2010; Chi, 2012). In this regard, rapamycin inhibits Cyclin D3 expression, which is upregulated during T cell activation due to mTOR signaling, but does not affect p27Kip1 levels, as demonstrated *in vitro* using E7 T cell lines and western blotting (Colombetti et al., 2006; Zheng et al., 2007). Hence, rapamycin treatment in CD4^+^ OT-II T cells significantly reduced their activation (measured as decreased CD44 expression) (Rao et al., 2010). Others have reported that most of rapamycin-sensitive CD4^+^ T cells in the presence of 100 mM Rapamycin indeed suffer from enhanced cell death (Battaglia et al., 2006). However, cells that survive rapamycin treatment proliferate even in the absence of exogenous IL-2 (Battaglia et al., 2006). These results are in line with the fact that rapamycin induces apoptosis in TCR-activated CD4^+^CD25^+^ conventional T cells, while natural T_regs_ are resistant to rapamycin-mediated apoptosis (Strauss et al., 2007). Here, rapamycin induced an upregulation of antiapoptotic Bcl-2 proteins in CD4^+^CD25^+^ T_regs_ (Strauss et al., 2009).

Similar to CD4^+^ T cells, rapamycin also modulates CD8^+^ T cell function. Rapamycin was reported to mediate the formation of memory CD8^+^ T cells *in vitro* (Araki et al., 2009; Pedicord et al., 2015; Moraschi et al., 2021). Furthermore, during vaccination of non-human primates, rapamycin enhanced CD8^+^ memory T cell responses in the expansion and contraction phase (Araki et al., 2009), which was associated with a loss of T-bet but a compensatory upregulation of the transcription factor Eomes, which regulates T-cell homeostasis and function (Rao et al., 2010; Figure 3). The combination of cytotoxic T-lymphocyte-associated protein 4 (CTLA-4) inhibitors and rapamycin increased the frequency of memory CD8^+^ T cells and improved memory response toward tumors and bacterial challenges (Pedicord et al., 2015). These results are in line with observations that a lack of mTORC2 signaling promotes CD8^+^ memory T cell function and that Rictor-deficient CD8^+^ T cells showed increased metabolic fitness (Pollizzi et al., 2015).

Rapamycin was shown to improve the effector and memory CD8^+^ T cell responses following immunization with the ASP2 protein of *Trypanosoma cruzi* (Moraschi et al., 2021). Here, rapamycin increased proliferation and cytotoxicity and stabilized effector, central memory, and effector memory CD8^+^ T cells. In addition, IFN-γ, TNF-α, and CD107a expression in CD8^+^ T cells appeared higher in the presence of rapamycin (Moraschi et al., 2021). Furthermore, rapamycin also increased the number of CD8^+^ virus-specific T cells after acute lymphocytic choriomeningitis (Araki et al., 2009). However, the exact mechanism remains unclear.

Taken together, rapamycin strongly interferes with T cell proliferation, differentiation, metabolism, and T cell functions. While rapamycin treatment of CD4 + T cells leads to robust T_reg_ differentiation, treatment of CD8 + T cells instead induces memory formation.

## Conclusion

Current literature shows that antibiotics preferentially exert suppressive rather than activating effects on CD4^+^ Th cells. While this may be potentially detrimental during infectious diseases, it could prove useful in the treatment of autoimmunity and allergic diseases. Regarding the latter, ciprofloxacin treatment resulted in a dose-dependent inhibition of TCR-induced IL-4 production of T cells from patients that suffer from atopic dermatitis (Kamiński et al., 2010).

In comparing the literature, it is clear that the effects of antibiotics on CD8^+^ T cells vary significantly. While, in general, antibiotics seem to reduce the effector functions of CD8^+^ T cells by interfering with proliferation and by inducing apoptosis, rapamycin selectively promotes the formation of memory CD8^+^ T cells. The latter may be beneficial especially during reinfection with the same pathogen. On the other hand, antibiotics may also interfere with antitumor immunity as demonstrated by the treatment of CD8^+^ T cells with tigecycline (OSullivan et al., 2021). In this regard, a clinical trial in patients with advanced non-small cell lung cancer (NSCLC) also revealed that antibiotic administration attenuates the efficacy of immune checkpoint inhibitors (Huemer et al., 2018). Here, it should be also mentioned that the usage of for instance rapamycin during antitumor therapy, would likely not only impair anti-tumor immunity but also evoke immunodeficiency and increase the risk of secondary infections. However, further studies on the administration of antibiotics during cancer therapy are needed to elucidate these mechanisms.

Current studies indicate that especially linezolid and rapamycin limit T cell-mediated autoimmunity (Seifert et al., 2018; Li et al., 2020; Almeida et al., 2021). In this context, linezolid was shown to impair central nervous system (CNS) autoimmunity by inhibiting the proliferation of self-reactive T cells during experimental autoimmune encephalitis (EAE), a mouse model for multiple sclerosis. Here, linezolid was shown to reduce the frequency of MOG-specific IL-17^+^ and IFN-γ^+^ T cells, whereas the frequency of T_regs_ appeared unchanged during CNS autoimmunity. It is of particular interest that the inhibitory effect of linezolid on Th1, Th2 and Th17 cells appeared without compromising the viability of Th cells (Almeida et al., 2021). However, to which extent linezolid also attenuates Th2 cell-mediated diseases like allergic asthma, atopic dermatitis, and allergic rhinitis, remains to be resolved.

Furthermore, administration of broad-spectrum antibiotics (ampicillin, metronidazole, neomycin, and vancomycin) were demonstrated to increase the frequencies of IL-10-producing regulatory B cells (CD19^+^CD138^+^CD44^hi^) and regulatory CD8^+^ cytotoxic T cells (CD8^+^CD122^+^) during EAE (Ochoa-Repáraz et al., 2009; Seifert et al., 2018). Rapamycin was identified to limit peripheral autoimmunity by promoting the expansion of T_regs_ and additionally by inhibiting Th17 cell responses. During EAE, rapamycin alleviated the disease course by promoting the TAM-TLRs-SOCS signaling pathway (Li et al., 2020). However, it should be mentioned that others reported that treatment with cefuroxime or other beta-lactam antibiotics leads to more aggravated EAE and adjuvant-induced arthritis due to directly increased T-cell pathogenicity and toxicity of these antibiotics (Mor and Cohen, 2013).

Taken together, based on numerous *in vitro* studies, mainly macrolides, fluoroquinolones and recently linezolid have been investigated as potential therapeutic agents in various allergic and autoimmune diseases due to their immunomodulatory properties independently of their anti-bacterial activity. Nevertheless, it should be critically considered that therapeutical use of antibiotics besides their antibacterial indication fosters the generation of multi-resistance germs creating a significant burden to our health care systems. Table 1 summarizes studies from the last 5 years, in which macrolides, phenicols, and oxazolidinones were tested in allergic and autoimmune diseases. Understanding the cellular and molecular effectors that contribute to the observed outcome of the individual studies will require prospective studies which investigate the effect of different antibiotics on T cells as well as innate immune cell populations during pathogenesis of such diseases.

**TABLE 1:** Studies investigating the effect of macrolides (red), phenicols (blue) and oxazolidinones (green) on allergic and autoimmune diseases.

Allergic diseases study	Antibiotic	Outcome
Park et al., 2020	Azithromycin	Alteration of gut microbiota may reduce airway inflammation in allergic asthmatic patients
Watts, 2017	Chloramphenicol	Severe delayed-type hypersensitivity (case report)
Ezeamuzie et al., 2022	Oxazolidinone hydroxamic acid derivative PH-251	Protection
Nam et al., 2018	Furaltadone	Protection
Phillips et al., 2020	Oxazolidinone hydroxamic acid derivatives	Anti-inflammatory activity *in vitro*
Yen et al., 2021	Erythromycin	Protection (decrease in IgE and IgG_2_)
Aquino and Rosner, 2019	Erythromycin	May cause systemic contact dermatitis
Lin et al., 2020	Macrolides	5-year antibiotic exposure to macrolides is associated with the risk of asthma development in allergic rhinitis children before age of 12
Sadamatsu et al., 2020	Non-antibiotic macrolide EM900	Protection
Yamamoto-Hanada et al., 2017	Macrolides a.o.	Exposure to antibiotics to participants ≤ 3 years revealed higher risk of developing allergic diseases at 5 years
Undela et al., 2021	Macrolides	Macrolides probably deliver a moderately sized reduction in exacerbations requiring hospitalizations compared to placebo (meta-analysis)

**Autoimmune diseases** **study**	**Antibiotic**	**Outcome**

Almeida et al., 2021	Linezolid	Protection
Grohmann et al., 2020	Linezolid	Induction of photoreceptor dysfunction which masquerades as autoimmune retinopathy (case report)
Huang et al., 2021	Azithromycin	Protection
Wang et al., 2018	Azithromycin	Protection?/Immune modulation
Tso et al., 2018	Clarithromycin	Protection (case report)
Ohe et al., 2018	Clarithromycin	Protection (case report)
Chen I. L. et al., 2021	Macrolides (especially Azithromycin)	Increased risk of new-onset asthma in children with atopic dermatitis
Drago et al., 2013; Butt et al., 2019; Rossi et al., 2021	Roxithromycin Clarithromycin Azithromycin	Association/Induction with/of acute autoimmune thrombocytopenia (case reports)

## Author contributions

SK designed the study. TF, JN, PB, LR, AK, AZ, and SK performed the literature search. TF, JN, PB, and SK drafted the manuscript. TF, JN, and SK designed the Figures 1–3. MB, MM-F, DM, AM, and AZ provided the critical academic input throughout the work and corrected the manuscript. All authors approved the final version of the manuscript.
